# Mechanical forces in lymphatic vessel development: Focus on transcriptional regulation

**DOI:** 10.3389/fphys.2022.1066460

**Published:** 2022-11-10

**Authors:** Naoto Ujiie, Tsutomu Kume

**Affiliations:** Department of Medicine, Feinberg School of Medicine, Feinberg Cardiovascular and Renal Research Institute, Northwestern University, Chicago, IL, United States

**Keywords:** lymphatic system, lymphatic vascular development, lymphangiogenesis, valve formation, transcriptional regulation

## Abstract

The lymphatic system is crucial for the maintenance of interstitial fluid and protein homeostasis. It has important roles in collecting excess plasma and interstitial fluid leaked from blood vessels, lipid absorption and transportation in the digestive system, and immune surveillance and response. The development of lymphatic vessels begins during fetal life as lymphatic endothelial progenitor cells first differentiate into lymphatic endothelial cells (LECs) by expressing the master lymphatic vascular regulator, prospero-related homeobox 1 (PROX1). The lymphatic vasculature forms a hierarchical network that consists of blind-ended and unidirectional vessels. Although much progress has been made in the elucidation of the cellular and molecular mechanisms underlying the formation of the lymphatic vascular system, the causes of lymphatic vessel abnormalities and disease are poorly understood and complicated; specifically, the mechanistic basis for transcriptional dysregulation in lymphatic vessel development remains largely unclear. In this review, we discuss the recent advances in our understanding of the molecular and cellular mechanisms of lymphatic vascular development, including LEC differentiation, lymphangiogenesis, and valve formation, and the significance of mechanical forces in lymphatic vessels, with a focus on transcriptional regulation. We also summarize the current knowledge on epigenetic mechanisms of lymphatic gene expression.

## Introduction

A well-organized lymphatic system including proper lymph fluid absorption and drainage is imperative for maintaining interstitial fluid and protein homeostasis. The lymphatic system is composed of a blind-ended, unidirectional network that contains absorptive vessels, primary lymphoid organs such as thymus and bone marrow, secondary lymphoid organs such as lymph nodes, spleen, and Peyer’s patches, and lymphoid tissues as adenoids and tonsils (Ruddle and Akirav, 2009; Choi et al., 2012). Lymphatic fluid is collected from the interstitial space into lymphatic capillaries, and these lymphatic vessels merge and gradually thicken as lymphatic collecting vessels. Lymph is eventually drained at the angulus venosus, which is the junction of the subclavian vein and internal jugular vein (Ruddle and Akirav, 2009; Choi et al., 2012; Randolph et al., 2017; Oliver et al., 2020). The lymphatic system also plays crucial roles in lipid absorption and transportation from the digestive tract to the blood circulation, as well as immune cell transport from the interstitium into the venous circulation (Alitalo et al., 2005; Tammela and Alitalo, 2010; Petrova and Koh, 2020; Landau et al., 2021). Lymphatic dysfunction causes interstitial fluid imbalance and edema, nutrient malabsorption, and inflammatory pathologies (Tammela and Alitalo, 2010; Saito et al., 2013; Abouelkheir et al., 2017). Lymphangiogenesis, the formation of new lymphatic vessels from the preexisting lymphatic vessels, relates to various diseases and pathologies such as lymphedema, tumor metastasis, and chronic inflammatory diseases including rheumatoid arthritis (Detmar and Hirakawa, 2002; Alitalo, 2011; Masood et al., 2022); however, the molecular mechanisms that regulate lymphatic endothelial cell proliferation and migration *via* transcriptional regulation remain largely unknown. In this review, we provide an update on the current knowledge regarding the development of the lymphatic vasculature and its mechanical force signals, especially focusing on transcriptional regulatory mechanisms.

## The development of the lymphatic vascular system

**FIGURE 1:**
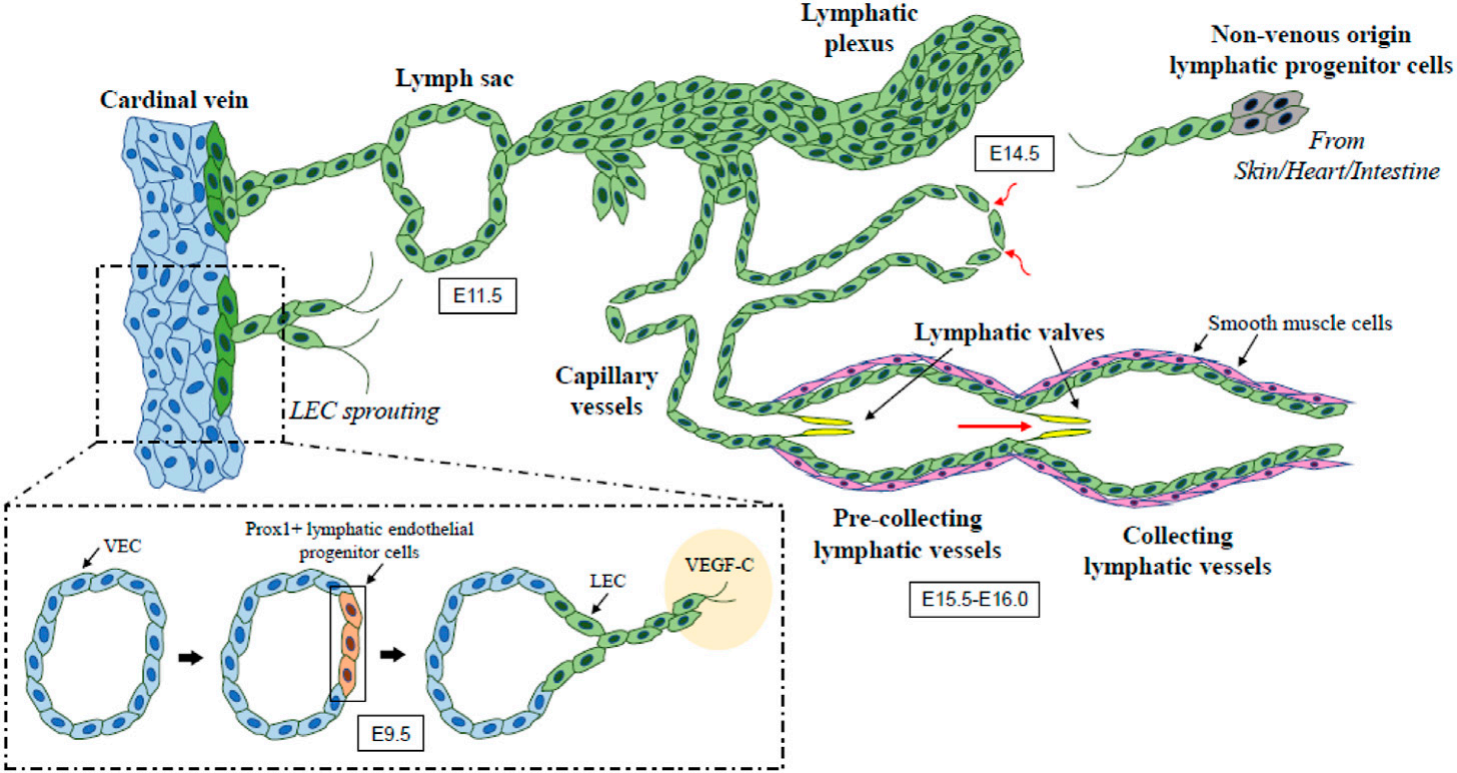
**The developmental process of lymphatic vessel**. Lymphatic endothelial progenitor cells in the cardinal vein begin to express *PROX1* (known as a master lymphatic vascular regulator) at E9.5 and then differentiate into LECs expressing *VEGFR3*. VEGF-C/VEGFR3 signaling promotes the sprouting and migration of *PROX1*-positive LECs, which leads to the formation of lymph sacs and initial lymphatic plexus by E11.5. Extended sprouting and migration of LECs from the initial lymphatic structures give rise to the hierarchical lymphatic vessel network. Non-venous origin lymphatic endothelial progenitor cells also contribute to the early lymphatic vasculature and the development of its network. This network undergoes remodeling and maturation during E15.5-E16.0 and forms the organized lymphatic vascular network including lymphatic capillaries and pre-collecting and collecting lymphatic vessels. Arrows show the direction of lymph flow. LEC, lymphatic endothelial cell; VEC, venous endothelial cell; VEGF, vascular endothelial growth factor.

The development of the lymphatic vascular system initiates shortly after blood circulation is established in mouse embryos (Yang and Oliver, 2014). At embryonic day (E) 9.5, a subpopulation of lymphatic endothelial progenitor cells in the anterior cardinal vein start to express the Prospero-related homeobox 1 (PROX1) transcription factor, which is a master lymphatic vascular regulator (Wigle and Oliver, 1999; Francois et al., 2008; Ducoli and Detmar, 2021), and then differentiate into lymphatic endothelial cells (LECs) (Lee et al., 2009; Yamazaki et al., 2009; Srinivasan et al., 2010; Srinivasan and Oliver, 2011; Escobedo and Oliver, 2016; Petrova and Koh, 2018). By around E10.0, *PROX1* positive lymphatic endothelial progenitor cells expressing vascular endothelial growth factor receptor (VEGFR) 3 sprout *via* stimulation with mesenchyme-derived VEGF-C ligand. These cells further migrate dorsolaterally from cardinal and intersomitic veins and establish primary lymph sacs and superficial lymphatic vessels identified as the jugular lymph sac by E11.5 (Wigle and Oliver, 1999; Karkkainen et al., 2004; Francois et al., 2012; Yang et al., 2012; Hagerling et al., 2013; Stritt et al., 2021). Another study also suggests LEC fate is decided during transition through the paraxial mesoderm (PXM) lineage. PXM-derived ECs selectively transdifferentiate from the cardinal vein to form LEC progenitors and form the lymphatic endothelium of multiple organs and tissues (Stone and Stainier, 2019). There is accumulating evidence that mesenchyme- or non-venous derived lymphatic progenitor cells contribute to the early lymphatic vasculature and the development of the lymphatic vascular network in various organs including the skin, heart, and mesentery (Bernier-Latmani et al., 2015; Klotz et al., 2015; Martinez-Corral et al., 2015; Stanczuk et al., 2015; Kazenwadel and Harvey, 2016; Ducoli and Detmar, 2021). This network spreads throughout the mouse embryo by E14.5 and subsequently goes through remodeling and maturation from E15.5-E16.0, forming the hierarchical structure of the lymphatic vascular network in which lymphatic capillaries merge to form pre-collecting and collecting lymphatic vessels (Coso et al., 2014; Norden and Kume, 2020) (Figure 1).

Lymphatic vessels are composed of lymphatic capillaries, which are also called initial lymphatics, and collecting lymphatic vessels. The basement membrane of lymphatic capillaries is discontinuous without lining of any pericytes or lymphatic smooth muscle cells (SMCs); therefore, they work for collecting excess plasma and interstitial fluid leaked from blood vessels (Norden and Kume, 2020). In contrast, collecting lymphatic vessels have lymphatic valves to prevent the backflow of lymph, and smooth muscle to transport lymph fluid by contraction (Kume, 2015). The development of the lymphatic vascular network is conducted by several critical signaling pathways including lymphangiogenic signaling such as the VEGF-C/D-VEGFR3 and Angiopoietin (Angpt)-tunica interna endothelial cell kinase (TEK, also known as Tie2) pathways (Potente and Makinen, 2017). Two transcription factors, the SRY-Box transcription factor 18 (SOX18) and the chick ovalbumin upstream promoter transcription factor 2 (COUP-TFII), also play an important role in lymphatic specification *via* the induction of *PROX1* expression, whereas different pathways such as Notch, retinoic acid, and Wnt/beta-catenin signaling are involved in this process (Nicenboim et al., 2015; Ducoli and Detmar, 2021). *VEGFR3* also regulates *PROX1* by establishing a feedback loop necessary to maintain the identity of LEC progenitor cells, and VEGF-C-mediated activation of Vegfr3 signaling is required to maintain *PROX1* expression in LEC progenitor cells (Srinivasan et al., 2014). In collecting lymphatic vessels, platelet-derived growth factor B (PDGFB) regulates lymphatic SMC recruitment, but PDGFB overexpression is insufficient to mediate recruitment to lymphatic capillaries (Wang et al., 2017).

A recent study has demonstrated the deficiency of Folliculin, a tumor suppressor, causes ectopic expression of *PROX1* in venous endothelial cells (VECs), leading to the misconnection of blood and lymphatic vessels (Tai-Nagara et al., 2020). In LEC-biased VECs deficient for Folliculin, the basic helix-loop-helix transcription factor E3 (TFE3) translocate into the nucleus, binds to a regulatory element of the *PROX1* gene, and induces its ectopic venous expression (Tai-Nagara et al., 2020). Thus, in mice, it has been shown that the transition of lymphatic specification and differentiation from venous cell fate is tightly controlled during development. Importantly, the development of the zebrafish anal fin begins along with the formation of lymphatic vessels, but not blood vessels. Following the progressive loss of lymphatic markers during the anal fin growth, these vessels subsequently acquire a blood vessel fate leading to the connection to blood circulation. Thus, this specialized blood vessel formation occurs through LEC transdifferentiation (Das et al., 2022). Single-cell RNA-sequencing analysis in this study further reveals that the loss of lymphatic fate results in the upregulation of several blood endothelial markers, such as *VEGFR1*, Delta-like (DLL) 4, and SRY-box (SOX) 17. Of note, mosaic overexpression of *SOX17* in zebrafish ECs results in reduced lymphatic gene expression in the anal fin as well as the absence or incomplete formation of the thoracic duct (Das et al., 2022), demonstrating the importance of *SOX17* function in the transition process.

### Transcriptional and epigenetic regulation in lymphangiogenesis

Transcriptional regulation during lymphangiogenesis is strictly controlled, and recent evidence suggests the specific functions of several key transcription factors in lymphangiogenesis. The transcription factor V-maf musculoaponeurotic fibrosarcoma oncogene homolog B (MAFB), which is involved in the differentiation of various cell types, regulates the transcriptional changes invoked by VEGF-C in LECs (Dieterich et al., 2015; Dieterich et al., 2020; Rondon-Galeano et al., 2020; Arnold et al., 2022). *MAFB* induces the expression of *PROX1*, other transcription factors and markers of differentiated LECs, indicating the role of *MAFB* in the maintenance of the mature LEC phenotype (Dieterich et al., 2015). LEC-specific *MAFB* deficiency in mice causes increased lymphatic branching in the diaphragm at P7, enhanced tumor-induced lymphangiogenesis, increased perinatal lethality associated with cyanosis, and excessive smooth muscle cell coverage indicating a defect in the maturation of lymphatic networks. This suggests *MAFB* could be a potential target for therapeutic modulation of lymphangiogenesis (Dieterich et al., 2020; Rondon-Galeano et al., 2020). The transcription factor hematopoietically expressed homeobox (HHEX), an upstream regulator of VEGF-C/VEGFR3/PROX1 signaling during angiogenic sprouting and lymphatic formation, is required cell-autonomously in endothelial cells to promote venous and lymphatic sprouting. Mice deficient for *HHEX* exhibit severe vascular defects in blood and lymphatic vessel development (Gauvrit et al., 2018).

Forkhead box (Fox) O1, a member of the Fox transcription factor family, acts an essential role in developmental lymphangiogenesis by promoting LEC migration toward the chemokine (C-X-C motif) ligand (CXCL) 12 and regulating their proliferative activity (Niimi et al., 2020). The LEC-specific deletion of *FOX O1* in mice decreases LEC migration toward CXCL12 by downregulating C-X-C chemokine receptor (CXCR) 4, induces excess LEC proliferation, and decreases LEC apoptosis, which leads to the disconnected and dilated structure of the lymphatic vessels (Niimi et al., 2020).

Brahma-related gene 1 (BRG1), a chromatin-remodeling enzyme, epigenetically regulates COUP-TFII expression, by remodeling chromatin within the *COUP-TFII* promoter and impacting the ability of transcriptional machinery to access the promoter (Davis et al., 2013). The EC-specific deletion of *Brg1* in mice results in downregulation of COUP-TFII expression in developing veins (Davis et al., 2013).

**FIGURE 2:**
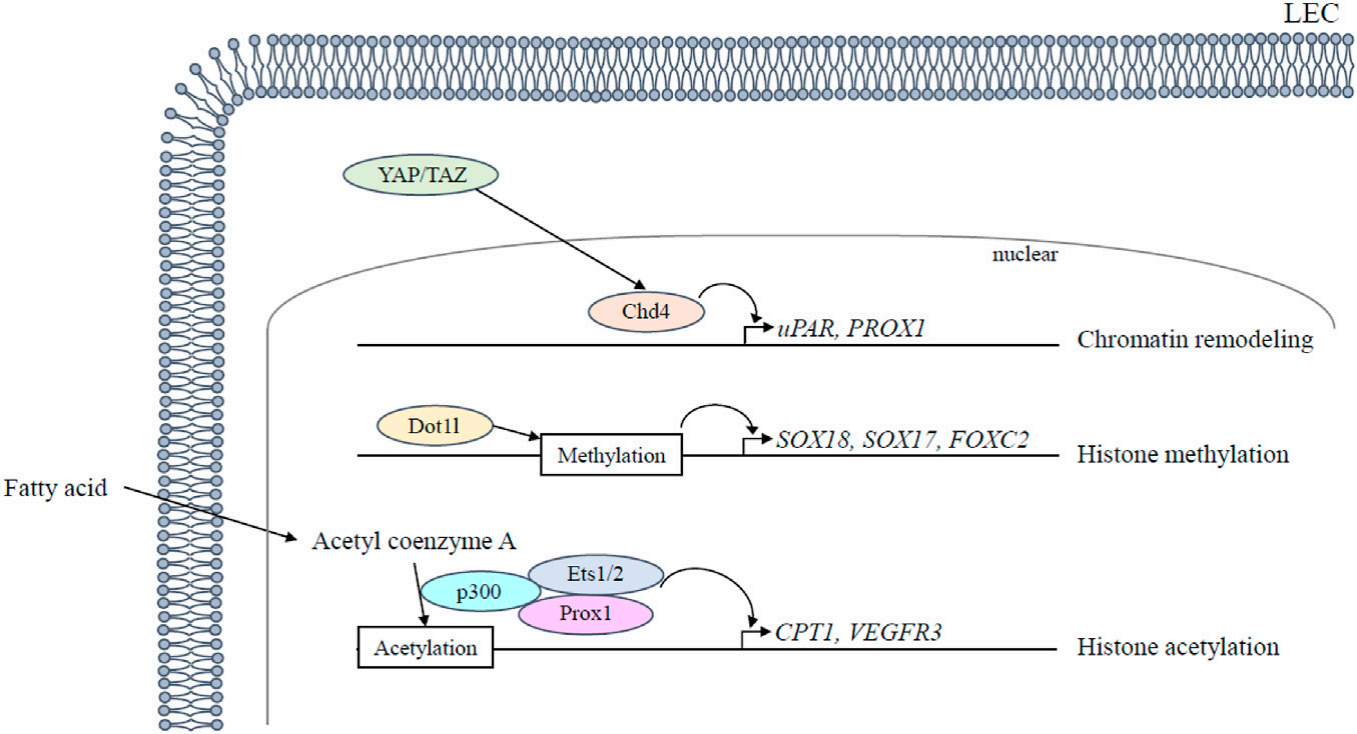
**Epigenetic regulation in lymphangiogenesis**. Epigenetic regulation regarding lymphatic development is mediated in LECs as follows: 1) *Chd4* is functionally associated with the Hippo signaling pathway and downregulates *Prox1* expression together with Hippo pathway final effectors *YAP/TAZ*; 2) *Dot1l* promotes transcription by histone methylation of chromatin; 3) Acetyl coenzyme A is used by the histone acetyltransferase p300 that interacts with *Prox1* to acetylate histones. ETS 1/2 participates in *VEGFR3* gene expression by recruiting the histone acetyltransferase p300 to the *VEGFR3* locus and leading to histone acetylation. LEC, lymphatic endothelial cell.

Chromodomain helicase DNA-binding 4 (CHD4), an ATPase subunit of the nucleosome remodeling deacetylase (NuRD) chromatin-remodeling complex, regulates vascular integrity in the mid-gestation (Ingram et al., 2013). Specifically, *CHD4* controls the development of lymphovenous valves, which regulates the return of lymph to the blood circulation by forming fibrin-rich thrombi that prevent blood from entering the lymphatic system (Crosswhite et al., 2016). The LEC-specific deletion of *CHD4* in mice leads to increased transcription of the urokinase plasminogen activator receptor (uPAR), thereby facilitating activation of the fibrin-degrading protease plasmin and then degrading the fibrin near the lymphovenous valves (Crosswhite et al., 2016). In addition, CDH4 is functionally associated with the Hippo signaling pathway in lymphatic endothelial cells (Figure 2). The Hippo pathway final effectors Yes-associated protein (YAP) and transcriptional coactivator with PDZ-binding motif (TAZ) promote remodeling of lymphatic plexus patterning and postnatal lymphatic valve maintenance by negatively regulating Prox1 expression (Cho et al., 2019). LEC-specific deletion of *YAP/TAZ* in mice suppresses both lymphatic plexus patterning and valve initiation *via* upregulation of *PROX1*, whereas LEC-specific *YAP/TAZ* overexpression downregulates *PROX1*, disrupts lymphatic specification, and restricts lymphatic sprouting (Cho et al., 2019).

Disruptor of telomeric silencing 1-like (DOT1L), a histone H3 lysine (H3K) 79 methyltransferase, promotes transcription by histone methylation of chromatin and is a crucial factor in the homeostasis of various organs such as the heart and hematopoiesis (Jo et al., 2011; Nguyen et al., 2011). Vascular endothelial cell (VEC)-specific, but not LEC-specific, deletion of *DOT1L* causes fully penetrant lymphatic aplasia by altering the lymphatic transcription program and reducing H3K79me2 enrichment at lymphatic genes, including the transcription factors *SOX18*, *SOX17*, and *FOXC2*, which are critical for LEC differentiation and valve formation, as well as Vegfr3, which is critical for LEC proliferation and migration (Yoo et al., 2020) (Figure 2).

LECs use fatty acid β-oxidation for proliferation and epigenetic regulation of *PROX1*, which mediates epigenetic changes that promote lymphangiogenesis during LEC differentiation (Figure 2): 1) *PROX1* upregulates carnitine palmitoyltransferase (CPT) 1A expression, which increases fatty acid β-oxidation-dependent acetyl coenzyme A production; 2) Acetyl coenzyme A is used by the histone acetyltransferase p300 to acetylate histones at lymphangiogenic genes; 3) histone acetyltransferase p300 interacts with Prox1 to facilitate preferential histone acetylation at the loci of *PROX1*-targeted genes (Wong et al., 2017). LEC-specific deletion of *CPT1A* in mice impairs lymphatic vessel development by exhibiting severe impairment of dermal lymphatic vessel outgrowth and branching at E16.5. (Wong et al., 2017). Other transcription factors expressed in LEC, E26 avian leukemia oncogene (ETS) 1 and 2, act as downstream effectors of the Ras/MAPK pathway and participate in *VEGFR3* gene expression in LECs by recruiting the histone acetyltransferase p300 to the *VEGFR3* locus and leading to histone acetylation and transcriptional activation of the *VEGFR3* promoter (Ichise et al., 2012). In addition, *ETS2* enhances inflammatory lymphangiogenesis and endothelial migration towards VEGF-C through the induction of *VEGFR3* expression by binding to the *VEGFR3* promoter in concert with *PROX1* (Yoshimatsu et al., 2011). Additionally, mitochondrial complex III also regulates the critical *PROX1*-*VEGFR3* feedback loop. The functional inactivation of mitochondrial complex III impairs lymphatic vessel development by disrupting the maintenance of the *PROX1*-*VEGFR3* feedback loop through the reduction in H3K4me3 and H3K27ac histone modifications at the *VEGFR3* and *PROX1* promoters (Ma et al., 2021).

### Transcriptional and epigenetic regulation in lymphatic valve formation

**FIGURE 3:**
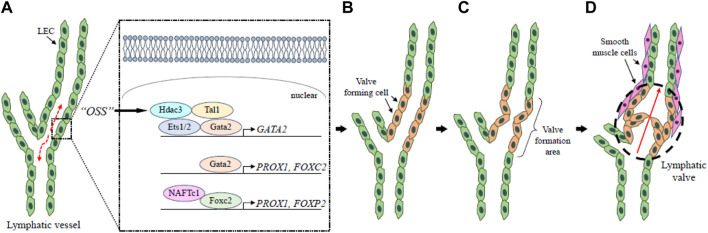
**Lymphatic valve formation**. The lymphatic valves are formed through lymph flow-dependent lymphatic vessel remodeling. At the bifurcation of lymphatic vessels, the flow of lymph generates OSS (broken arrows) that leads to the upregulation of *GATA2* in LECs *via* Hdac3 **(A)**. Gata2 regulates *PROX1* and *FOXC2* expression to establish valve-forming cells **(B)**. Following these processes, the valve formation area is determined **(C)**, and the lymphatic valve (broken circle) has a role in keeping the lymph flow unidirectional **(D)**. Red arrow shows the direction of lymph flow. LEC, lymphatic endothelial cell; OSS, oscillatory share stress.

Lymph flow is essential for the development and maturation of lymphatic valves (Kume, 2015), which play a critical role in preventing the backflow of lymph fluid. The lymphatic valves are formed from lymphatic endothelial cells, a process that is occurred by flow-dependent lymphatic vessel remodeling caused by oscillatory share stress (OSS) at branching points in the lymphatic plexus during the early stage of lymphatic development (Sabine et al., 2012; Shin and Lawson, 2021). The OSS response leads to an increase in the expression of GATA-binding protein 2 (GATA2), Prox1, and Foxc2, which induce valve forming cells to the site of valve formation (Kazenwadel et al., 2015; Sweet et al., 2015; Shin and Lawson, 2021). In valve forming cells, Gata2 directly regulates *PROX1* and *FOXC2* expression, whereas Foxc2 regulates valve maturation in cooperation with Prox1 to control intraluminal invagination of LECs and reorganization into valve forming leaflets by postnatal day (P)1 (Kazenwadel et al., 2015). As an upstream epigenetic factor, the histone-modifying enzyme histone deacetylase 3 (HDAC3) regulates lymphatic valve formation. In response to OSS, Hdac3 is recruited to the Gata2 enhancer element and physically interacts with the transcription factors T-cell acute lymphocytic leukemia protein 1 (TAL1), Gata2, and Ets1/2 to promote Gata2 expression (Janardhan et al., 2017) (Figure 3).

Human *FOXC2* is a causative gene whose mutations are dominantly associated with lymphedema-distichiasis syndrome characterized by failure of lymph drainage in limbs, venous valve failure, and the growth of an extra set of eyelashes (Fang et al., 2000; Traboulsi et al., 2002; Mellor et al., 2007; Tavian et al., 2016). Foxc2 regulates connexin 37 expression and activation of calcineurin/nuclear factor of activated T-cells (NFAT) signaling during lymphatic collecting vessel maturation and valve formation (Petrova et al., 2004; Norrmen et al., 2009; Sabine et al., 2012). Foxc2 is also identified as a crucial factor for lymphatic valve maintenance by regulating LEC junctional integrity and cellular quiescence under reversing flow conditions *via* restriction of TAZ-mediated proliferation (Sabine et al., 2015). Foxc1 is a closely related member of the Fox transcription factor family, and LEC-specific deletion of *FOXC1*, *FOXC2*, or both in mice leads to increased LEC proliferation, enlarged lymphatic vessels, and abnormal lymphatic vessel morphogenesis, accompanied by increased Ras/ERK signaling during embryonic lymphangiogenesis (Fatima et al., 2016). Unlike *FOXC2*, LEC-specific *FOXC1* mutant mice normally develop initial mesenteric lymphatic valves; however, the formation of matured lymphatic vessels (v-shaped or semilunar bi-leaflet structures) is significantly impaired (Norden et al., 2020). Importantly, the number of mesenteric lymphatic valves is remarkably reduced in the LEC-specific deletion of *FOXC1* and *FOXC2* compared to LEC-specific *FOXC2* deletion alone, suggesting that *FOXC1* and *FOXC2* function cooperatively in the maturation and maintenance of lymphatic valves (Norden et al., 2020).

Foxo1 is crucial for controlling the expression of valve forming genes including *FOXC2*, *PROX1*, and *GATA2* as a key downstream effector of shear stress by regulating lymphatic valve maintenance, and LEC-specific deletion of *FOXO1* in mice leads to the formation of additional lymphatic valves compared to control mice (Scallan et al., 2021). Another study also reveals the role of Foxo1 as a repressor for lymphatic valve formation and maintenance *via* the inhibition of OSS-induced upregulation of lymphatic valve-specific genes such as *PROX1* and *FOXC2* (Niimi et al., 2021).

A recent study has shown that Foxp2, another Fox transcription factor previously implicated in speech development, is expressed in lymphatic endothelial cells of collecting vessels and their valve-forming cells, and that Foxp2 is induced after initiation of lymph flow and upon OSS on LECs (Hernandez Vasquez et al., 2021). LEC-specific *FOXP2* mutant mice exhibit enlarged collecting vessels and defective lymphatic valves characterized by loss of NFATc1 activity (Hernandez Vasquez et al., 2021).

Piezo type mechanosensitive ion channel component 1 (PIEZO1), a cation channel activated by mechanical forces such as fluid shear stress or membrane stretch, is a causative gene associated with congenital lymphedema with pleural effusion (Nonomura et al., 2018). The LEC-specific deletion of *PIEZO1* in mice leads to a reduction in the number of lymphatic valves and impairments in lymphatic valve protrusion such as collective cell migration, actin polymerization, and remodeling of cell-cell junctions, whereas the expression patterns of Foxc2 and NFATc1, both of which are crucial factors for lymphatic valve development, are normally detected in these mutant mice (Nonomura et al., 2018). Another study demonstrated that PIEZO1 is the force sensor in the mechanotransduction pathway controlling lymphatic valve development and maintenance, although *PIEZO1* knockdown in cultured LECs largely abrogated the OSS-induced upregulation of the lymphatic valve signature genes including *FOXC2* and *GATA2* (Choi et al., 2019). Moreover, overexpressing *PIEZO1* in cultured LECs upregulates *FOXC2* and *GATA2* in the absence of OSS, demonstrating that ectopic expression of *PIEZO1* can recapitulate the lymphatic valve gene profile (Choi et al., 2019). Treatment with Yoda1, a chemical agonist of PIEZO1, leads to changes in LEC morphology by inducing the remodeling of actomyosin and/or VE-cadherin^+^ cell–cell adhesion and activates the expression of some lymphatic valve genes such as *FOXC2* and *GATA2* in a PIEZO1-dependent manner (Nonomura et al., 2018; Choi et al., 2019). Together, these results suggest that the activation of mechanosensitive PIEZO1 can control, at least in part, transcriptional regulation of lymphatic valve forming cells under the absence of mechanical forces.

## Concluding remarks

Many studies have been conducted on the formation, maintenance, and function of lymphatic vessels, which are essential to maintain homeostasis. This review focuses on the mechanisms of transcriptional regulation in LECs during lymphatic vessel development (Table 1), but the precise control of lymphatic gene expression in lymphangiogenesis under physiological and pathological conditions remains unexplored. Recent single-cell RNA-sequencing studies using LEC markers such as *PROX1* and *VEGFR3* have started to clarify LEC heterogeneity in various organs including functional multiformity. For example, single-cell RNA-sequencing analysis using the zebrafish anal fin was the key to characterizing the different endothelial cell populations and transition states involved in the LEC transdifferentiation process (Das et al., 2022). Moreover, single-cell transcriptomic analysis of normal and glaucomatous Angpt1 deficient eyes has recently identified distinct trabecular meshwork (TM) and Schlemm’s canal (SC) cell populations and revealed additional TM-SC signaling pathways (Thomson et al., 2021). Yet, additional comprehensive studies are needed to fully elucidate the mechanisms of transcriptional regulation of LECs with which the signaling pathways are associated. In particular, uncovering the transcriptional mechanisms underlying lymphangiogenesis will likely lead to the development of new therapeutic strategies for various diseases regarding lymphatic vessels.

**TABLE 1:** Factors involved in transcriptional regulation regarding lymphatic vascular development.

Factor	Function	References
*PROX1*	A master lymphatic vascular regulator	
	Promotes differentiation of lymphatic endothelial progenitor cells in the cardinal vein into LECs	Wigle and Oliver, (1999)	
	
		Francois et al. (2008)
		Lee et al. (2009)
		Yamazaki et al. (2009)
		Srinivasan et al. (2010)
		Srinivasan and Oliver, (2011)
		Escobedo and Oliver, (2016)
		Petrova and Koh, (2018)
		Ducoli and Detmar, (2021)
*MAFB*	Induction of Prox1 expression in differentiated LECs	Dieterich et al. (2015)
	Maintenance of mature LEC phenotype	
*FOXO1*	Acts an essential role in normal developmental lymphangiogenesis by promoting LEC migration toward CXCL12 and regulating their proliferative activity	
	Control the expression of valve forming genes including *FOXC2*, *PROX1*, and *GATA2*		
	Repressor for lymphatic valve formation and maintenance	Niimi et al. (2020)	
	
		Scallan et al. (2021)
*HHEX*	Promotes venous and lymphatic sprouting	Gauvrit et al. (2018)
*CHD4*	Acts normal lymphovenous valve development	
	Regulates the return of lymph to the blood circulation by forming fibrin-rich thrombi that prevent blood from entering the lymphatic system	Crosswhite et al. (2016)	
*YAP/TAZ*	Promotes remodeling lymphatic plexus patterning and postnatal lymphatic valve maintenance	Cho et al. (2019)
*DOT1L*	Promotes transcription by histone methylation of chromatin and promotes the expression of important transcription factors such as Sox18, Foxc2, and VEGFR3 in lymphatic endothelium	Yoo et al. (2020)
*GATA2*	Induces valve forming cells to the site of valve formation	Kazenwadel et al. (2015)
	Directly regulates *PROX1* and *FOXC2* expression in valve forming cells	
*FOXC1/FOXC2*	Required for LEC junction integrity in lymphatic valves, collecting vessels, and dermal lymphatics	Petrova et al. (2004)
		Norrmen et al. (2009)
		Sabine et al. (2015)
		Fatima et al. (2016)
		Norden et al. (2020)
*FOXP2*	Maintenance of collecting lymphatic vessel and valve formation	Hernandez Vasquez et al. (2021)
*PIEZO1*	Maintenance of the lymphatic valve protrusion such as collective cell migration, actin polymerization, and remodeling of cell-cell junctions	
	Upregulates *FOXC2* and *GATA2* under the absence of OSS in valve forming cells	Nonomura et al. (2018)	
		Choi et al. (2019)

LEC, lymphatic endothelial cell.

## References

[B1] Abouelkheir G. R. Upchurch B. D. Rutkowski J. M. (2017). Lymphangiogenesis: Fuel, smoke, or extinguisher of inflammation's fire? Exp. Biol. Med. 242, 884–895. 10.1177/1535370217697385 PMC5407543 28346012

[B2] Alitalo K. Tammela T. Petrova T. V. (2005). Lymphangiogenesis in development and human disease. Nature 438, 946–953. 10.1038/nature04480 16355212

[B3] Alitalo K. (2011). The lymphatic vasculature in disease. Nat. Med. 17, 1371–1380. 10.1038/nm.2545 22064427

[B4] Arnold H. Panara V. Hussmann M. Filipek-Gorniok B. Skoczylas R. Ranefall P. (2022). Mafba and mafbb differentially regulate lymphatic endothelial cell migration in topographically distinct manners. Cell Rep. 39, 110982. 10.1016/j.celrep.2022.110982 35732122

[B5] Bernier-Latmani J. Sabine A. Petrova T. V. (2015). Meet me in the middle: Dual origins of dermal lymphatic vasculature in mammals. Circ. Res. 116, 1630–1632. 10.1161/CIRCRESAHA.115.306436 25953918

[B6] Cho H. Kim J. Ahn J. H. Hong Y. K. Makinen T. Lim D. S. (2019). YAP and TAZ negatively regulate Prox1 during developmental and pathologic lymphangiogenesis. Circ. Res. 124, 225–242. 10.1161/CIRCRESAHA.118.313707 30582452

[B7] Choi D. Park E. Jung E. Cha B. Lee S. Yu J. (2019). Piezo1 incorporates mechanical force signals into the genetic program that governs lymphatic valve development and maintenance. JCI Insight 4, 125068. 10.1172/jci.insight.125068 30676326 PMC6483520

[B8] Choi I. Lee S. Hong Y. K. (2012). The new era of the lymphatic system: No longer secondary to the blood vascular system. Cold Spring Harb. Perspect. Med. 2, a006445. 10.1101/cshperspect.a006445 22474611 PMC3312397

[B9] Coso S. Bovay E. Petrova T. V. (2014). Pressing the right buttons: Signaling in lymphangiogenesis. Blood 123, 2614–2624. 10.1182/blood-2013-12-297317 24608974

[B10] Crosswhite P. L. Podsiadlowska J. J. Curtis C. D. Gao S. Xia L. Srinivasan R. S. (2016). CHD4-regulated plasmin activation impacts lymphovenous hemostasis and hepatic vascular integrity. J. Clin. 126, 2254–2266. 10.1172/JCI84652 PMC4887170 27140400

[B11] Das R. N. Tevet Y. Safriel S. Han Y. Moshe N. Lambiase G. (2022). Generation of specialized blood vessels via lymphatic transdifferentiation. Nature 606, 570–575. 10.1038/s41586-022-04766-2 35614218 PMC9875863

[B12] Davis R. B. Curtis C. D. Griffin C. T. (2013). BRG1 promotes COUP-TFII expression and venous specification during embryonic vascular development. Development 140, 1272–1281. 10.1242/dev.087379 23406903 PMC3585661

[B13] Detmar M. Hirakawa S. (2002). The formation of lymphatic vessels and its importance in the setting of malignancy. J. Exp. Med. 196, 713–718. 10.1084/jem.20021346 12235205 PMC2194053

[B14] Dieterich L. C. Klein S. Mathelier A. Sliwa-Primorac A. Ma Q. Hong Y. K. (2015). DeepCAGE transcriptomics reveal an important role of the transcription factor MAFB in the lymphatic endothelium. Cell Rep. 13, 1493–1504. 10.1016/j.celrep.2015.10.002 26549461

[B15] Dieterich L. C. Tacconi C. Menzi F. Proulx S. T. Kapaklikaya K. Hamada M. (2020). Lymphatic MAFB regulates vascular patterning during developmental and pathological lymphangiogenesis. Angiogenesis 23, 411–423. 10.1007/s10456-020-09721-1 32307629 PMC7311381

[B16] Ducoli L. Detmar M. (2021). Beyond PROX1: Transcriptional, epigenetic, and noncoding RNA regulation of lymphatic identity and function. Dev. Cell 56, 406–426. 10.1016/j.devcel.2021.01.018 33621491

[B17] Escobedo N. Oliver G. (2016). Lymphangiogenesis: Origin, specification, and cell fate determination. Annu. Rev. Cell Dev. Biol. 32, 677–691. 10.1146/annurev-cellbio-111315-124944 27298093

[B18] Fang J. Dagenais S. L. Erickson R. P. Arlt M. F. Glynn M. W. Gorski J. L. (2000). Mutations in FOXC2 (MFH-1), a forkhead family transcription factor, are responsible for the hereditary lymphedema-distichiasis syndrome. Am. J. Hum. Genet. 67, 1382–1388. 10.1086/316915 11078474 PMC1287915

[B19] Fatima A. Wang Y. Uchida Y. Norden P. Liu T. Culver A. (2016). Foxc1 and Foxc2 deletion causes abnormal lymphangiogenesis and correlates with ERK hyperactivation. J. Clin. Invest. 126, 2437–2451. 10.1172/JCI80465 27214551 PMC4922698

[B20] Francois M. Caprini A. Hosking B. Orsenigo F. Wilhelm D. Browne C. (2008). Sox18 induces development of the lymphatic vasculature in mice. Nature 456, 643–647. 10.1038/nature07391 18931657

[B21] Francois M. Short K. Secker G. A. Combes A. Schwarz Q. Davidson T. L. (2012). Segmental territories along the cardinal veins generate lymph sacs via a ballooning mechanism during embryonic lymphangiogenesis in mice. Dev. Biol. 364, 89–98. 10.1016/j.ydbio.2011.12.032 22230615

[B22] Gauvrit S. Villasenor A. Strilic B. Kitchen P. Collins M. M. Marin-Juez R. (2018). HHEX is a transcriptional regulator of the VEGFC/FLT4/PROX1 signaling axis during vascular development. Nat. Commun. 9, 2704. 10.1038/s41467-018-05039-1 30006544 PMC6045644

[B23] Hagerling R. Pollmann C. Andreas M. Schmidt C. Nurmi H. Adams R. H. (2013). A novel multistep mechanism for initial lymphangiogenesis in mouse embryos based on ultramicroscopy. EMBO J. 32, 629–644. 10.1038/emboj.2012.340 23299940 PMC3590982

[B24] Hernandez Vasquez M. N. Ulvmar M. H. Gonzalez-Loyola A. Kritikos I. Sun Y. He L. (2021). Transcription factor FOXP2 is a flow-induced regulator of collecting lymphatic vessels. EMBO J. 40, e107192. 10.15252/embj.2020107192 33934370 PMC8204859

[B25] Ichise T. Yoshida N. Ichise H. (2012). Ras/MAPK signaling modulates VEGFR-3 expression through Ets-mediated p300 recruitment and histone acetylation on the Vegfr3 gene in lymphatic endothelial cells. PLoS One 7, e51639. 10.1371/journal.pone.0051639 23284731 PMC3524184

[B26] Ingram K. G. Curtis C. D. Silasi-Mansat R. Lupu F. Griffin C. T. (2013). The NuRD chromatin-remodeling enzyme CHD4 promotes embryonic vascular integrity by transcriptionally regulating extracellular matrix proteolysis. PLoS Genet. 9, e1004031. 10.1371/journal.pgen.1004031 24348274 PMC3861115

[B27] Janardhan H. P. Milstone Z. J. Shin M. Lawson N. D. Keaney J. F. Jr. Trivedi C. M. (2017). Hdac3 regulates lymphovenous and lymphatic valve formation. J. Clin. Invest. 127, 4193–4206. 10.1172/JCI92852 29035278 PMC5663362

[B28] Jo S. Y. Granowicz E. M. Maillard I. Thomas D. Hess J. L. (2011). Requirement for Dot1l in murine postnatal hematopoiesis and leukemogenesis by MLL translocation. Blood 117, 4759–4768. 10.1182/blood-2010-12-327668 21398221 PMC3100687

[B29] Karkkainen M. J. Haiko P. Sainio K. Partanen J. Taipale J. Petrova T. V. (2004). Vascular endothelial growth factor C is required for sprouting of the first lymphatic vessels from embryonic veins. Nat. Immunol. 5, 74–80. 10.1038/ni1013 14634646

[B30] Kazenwadel J. Betterman K. L. Chong C. E. Stokes P. H. Lee Y. K. Secker G. A. (2015). GATA2 is required for lymphatic vessel valve development and maintenance. J. Clin. 125, 2979–2994. 10.1172/JCI78888 PMC4563742 26214525

[B31] Kazenwadel J. Harvey N. L. (2016). Morphogenesis of the lymphatic vasculature: A focus on new progenitors and cellular mechanisms important for constructing lymphatic vessels. Dev. Dyn. 245, 209–219. 10.1002/dvdy.24313 26228815

[B32] Klotz L. Norman S. Vieira J. M. Masters M. Rohling M. Dube K. N. (2015). Cardiac lymphatics are heterogeneous in origin and respond to injury. Nature 522, 62–67. 10.1038/nature14483 25992544 PMC4458138

[B33] Kume T. (2015). Lymphatic vessel development: Fluid flow and valve-forming cells. J. Clin. 125, 2924–2926. 10.1172/JCI83189 PMC4563766 26214518

[B34] Landau S. Newman A. Edri S. Michael I. Ben-Shaul S. Shandalov Y. (2021). Investigating lymphangiogenesis *in vitro* and *in vivo* using engineered human lymphatic vessel networks. Proc. Natl. Acad. Sci. U. S. A. 118, e2101931118. 10.1073/pnas.2101931118 34326257 PMC8346860

[B35] Lee S. Kang J. Yoo J. Ganesan S. K. Cook S. C. Aguilar B. (2009). Prox1 physically and functionally interacts with COUP-TFII to specify lymphatic endothelial cell fate. Blood 113, 1856–1859. 10.1182/blood-2008-03-145789 18815287 PMC2647678

[B36] Ma W. Gil H. J. Liu X. Diebold L. P. Morgan M. A. Oxendine-Burns M. J. (2021). Mitochondrial respiration controls the Prox1-Vegfr3 feedback loop during lymphatic endothelial cell fate specification and maintenance. Sci. Adv. 7, eabe7359. 10.1126/sciadv.abe7359 33931446 PMC8087398

[B37] Martinez-Corral I. Ulvmar M. H. Stanczuk L. Tatin F. Kizhatil K. John S. W. (2015). Nonvenous origin of dermal lymphatic vasculature. Circ. Res. 116, 1649–1654. 10.1161/CIRCRESAHA.116.306170 25737499

[B38] Masood F. Bhattaram R. Rosenblatt M. I. Kazlauskas A. Chang J. H. Azar D. T. (2022). Lymphatic vessel regression and its therapeutic applications: Learning from principles of blood vessel regression. Front. Physiol. 13, 846936. 10.3389/fphys.2022.846936 35392370 PMC8980686

[B39] Mellor R. H. Brice G. Stanton A. W. French J. Smith A. Jeffery S. (2007). Mutations in FOXC2 are strongly associated with primary valve failure in veins of the lower limb. Circulation 115, 1912–1920. 10.1161/CIRCULATIONAHA.106.675348 17372167

[B40] Nguyen A. T. Xiao B. Neppl R. L. Kallin E. M. Li J. Chen T. (2011). DOT1L regulates dystrophin expression and is critical for cardiac function. Genes Dev. 25, 263–274. 10.1101/gad.2018511 21289070 PMC3034901

[B41] Nicenboim J. Malkinson G. Lupo T. Asaf L. Sela Y. Mayseless O. (2015). Lymphatic vessels arise from specialized angioblasts within a venous niche. Nature 522, 56–61. 10.1038/nature14425 25992545

[B42] Niimi K. Kohara M. Sedoh E. Fukumoto M. Shibata S. Sawano T. (2020). FOXO1 regulates developmental lymphangiogenesis by upregulating CXCR4 in the mouse-tail dermis. Development 147 (2), dev181545. 10.1242/dev.181545 31852686

[B43] Niimi K. Nakae J. Inagaki S. Furuyama T. (2021). FOXO1 represses lymphatic valve formation and maintenance via PRDM1. Cell Rep. 37, 110048. 10.1016/j.celrep.2021.110048 34852224

[B44] Nonomura K. Lukacs V. Sweet D. T. Goddard L. M. Kanie A. Whitwam T. (2018). Mechanically activated ion channel PIEZO1 is required for lymphatic valve formation. Proc. Natl. Acad. Sci. U. S. A. 115, 12817–12822. 10.1073/pnas.1817070115 30482854 PMC6294938

[B45] Norden P. R. Kume T. (2020). Molecular mechanisms controlling lymphatic endothelial junction integrity. Front. Cell Dev. Biol. 8, 627647. 10.3389/fcell.2020.627647 33521001 PMC7841202

[B46] Norden P. R. Sabine A. Wang Y. Demir C. S. Liu T. Petrova T. V. (2020). Shear stimulation of FOXC1 and FOXC2 differentially regulates cytoskeletal activity during lymphatic valve maturation. Elife 9, e53814. 10.7554/eLife.53814 32510325 PMC7302880

[B47] Norrmen C. Ivanov K. I. Cheng J. Zangger N. Delorenzi M. Jaquet M. (2009). FOXC2 controls formation and maturation of lymphatic collecting vessels through cooperation with NFATc1. J. Cell Biol. 185, 439–457. 10.1083/jcb.200901104 19398761 PMC2700385

[B48] Oliver G. Kipnis J. Randolph G. J. Harvey N. L. (2020). The lymphatic vasculature in the 21(st) century: Novel functional roles in homeostasis and disease. Cell 182, 270–296. 10.1016/j.cell.2020.06.039 32707093 PMC7392116

[B49] Petrova T. V. Karpanen T. Norrmen C. Mellor R. Tamakoshi T. Finegold D. (2004). Defective valves and abnormal mural cell recruitment underlie lymphatic vascular failure in lymphedema distichiasis. Nat. Med. 10, 974–981. 10.1038/nm1094 15322537

[B50] Petrova T. V. Koh G. Y. (2020). Biological functions of lymphatic vessels. Science 369, eaax4063. 10.1126/science.aax4063 32646971

[B51] Petrova T. V. Koh G. Y. (2018). Organ-specific lymphatic vasculature: From development to pathophysiology. J. Exp. Med. 215, 35–49. 10.1084/jem.20171868 29242199 PMC5748863

[B52] Potente M. Makinen T. (2017). Vascular heterogeneity and specialization in development and disease. Nat. Rev. Mol. Cell Biol. 18, 477–494. 10.1038/nrm.2017.36 28537573

[B53] Randolph G. J. Ivanov S. Zinselmeyer B. H. Scallan J. P. (2017). The lymphatic system: Integral roles in immunity. Annu. Rev. Immunol. 35, 31–52. 10.1146/annurev-immunol-041015-055354 27860528 PMC5551392

[B54] Rondon-Galeano M. Skoczylas R. Bower N. I. Simons C. Gordon E. Francois M. (2020). MAFB modulates the maturation of lymphatic vascular networks in mice. Dev. Dyn. 249, 1201–1216. 10.1002/dvdy.209 32525258

[B55] Ruddle N. H. Akirav E. M. (2009). Secondary lymphoid organs: Responding to genetic and environmental cues in ontogeny and the immune response. J. Immunol. 183, 2205–2212. 10.4049/jimmunol.0804324 19661265 PMC2766168

[B56] Sabine A. Agalarov Y. Maby-El Hajjami H. Jaquet M. Hagerling R. Pollmann C. (2012). Mechanotransduction, PROX1, and FOXC2 cooperate to control connexin37 and calcineurin during lymphatic-valve formation. Dev. Cell 22, 430–445. 10.1016/j.devcel.2011.12.020 22306086

[B57] Sabine A. Bovay E. Demir C. S. Kimura W. Jaquet M. Agalarov Y. (2015). FOXC2 and fluid shear stress stabilize postnatal lymphatic vasculature. J. Clin. 125, 3861–3877. 10.1172/JCI80454 PMC4607114 26389677

[B58] Saito Y. Nakagami H. Kaneda Y. Morishita R. (2013). Lymphedema and therapeutic lymphangiogenesis. Biomed. Res. Int. 2013, 804675. 10.1155/2013/804675 24222916 PMC3810055

[B59] Scallan J. P. Knauer L. A. Hou H. Castorena-Gonzalez J. A. Davis M. J. Yang Y. (2021). Foxo1 deletion promotes the growth of new lymphatic valves. J. Clin. 131, 142341. 10.1172/JCI142341 PMC8279588 34263740

[B60] Shin M. Lawson N. D. (2021). Back and forth: History of and new insights on the vertebrate lymphatic valve. Dev. Growth Differ. 63, 523–535. 10.1111/dgd.12757 34716915 PMC9299638

[B61] Srinivasan R. S. Escobedo N. Yang Y. Interiano A. Dillard M. E. Finkelstein D. (2014). The Prox1-Vegfr3 feedback loop maintains the identity and the number of lymphatic endothelial cell progenitors. Genes Dev. 28, 2175–2187. 10.1101/gad.216226.113 25274728 PMC4180978

[B62] Srinivasan R. S. Geng X. Yang Y. Wang Y. Mukatira S. Studer M. (2010). The nuclear hormone receptor Coup-TFII is required for the initiation and early maintenance of Prox1 expression in lymphatic endothelial cells. Genes Dev. 24, 696–707. 10.1101/gad.1859310 20360386 PMC2849126

[B63] Srinivasan R. S. Oliver G. (2011). Prox1 dosage controls the number of lymphatic endothelial cell progenitors and the formation of the lymphovenous valves. Genes Dev. 25, 2187–2197. 10.1101/gad.16974811 22012621 PMC3205588

[B64] Stanczuk L. Martinez-Corral I. Ulvmar M. H. Zhang Y. Lavina B. Fruttiger M. (2015). cKit lineage hemogenic endothelium-derived cells contribute to mesenteric lymphatic vessels. Cell Rep. 10, 1708–1721. 10.1016/j.celrep.2015.02.026 25772358

[B65] Stone O. A. Stainier D. Y. R. (2019). Paraxial mesoderm is the major source of lymphatic endothelium. Dev. Cell 50, 247–255. 10.1016/j.devcel.2019.04.034 31130354 PMC6658618

[B66] Stritt S. Koltowska K. Makinen T. (2021). Homeostatic maintenance of the lymphatic vasculature. Trends Mol. Med. 27, 955–970. 10.1016/j.molmed.2021.07.003 34332911

[B67] Sweet D. T. Jimenez J. M. Chang J. Hess P. R. Mericko-Ishizuka P. Fu J. (2015). Lymph flow regulates collecting lymphatic vessel maturation *in vivo* . J. Clin. Invest. 125, 2995–3007. 10.1172/JCI79386 26214523 PMC4563745

[B68] Tai-Nagara I. Hasumi Y. Kusumoto D. Hasumi H. Okabe K. Ando T. (2020). Blood and lymphatic systems are segregated by the FLCN tumor suppressor. Nat. Commun. 11, 6314. 10.1038/s41467-020-20156-6 33298956 PMC7725783

[B69] Tammela T. Alitalo K. (2010). Lymphangiogenesis: Molecular mechanisms and future promise. Cell 140, 460–476. 10.1016/j.cell.2010.01.045 20178740

[B70] Tavian D. Missaglia S. Maltese P. E. Michelini S. Fiorentino A. Ricci M. (2016). FOXC2 disease-mutations identified in lymphedema-distichiasis patients cause both loss and gain of protein function. Oncotarget 7, 54228–54239. 10.18632/oncotarget.9797 27276711 PMC5342337

[B71] Thomson B. R. Liu P. Onay T. Du J. Tompson S. W. Misener S. (2021). Cellular crosstalk regulates the aqueous humor outflow pathway and provides new targets for glaucoma therapies. Nat. Commun. 12, 6072. 10.1038/s41467-021-26346-0 34663817 PMC8523664

[B72] Traboulsi E. I. Al-Khayer K. Matsumoto M. Kimak M. A. Crowe S. Wilson S. E. (2002). Lymphedema-distichiasis syndrome and FOXC2 gene mutation. Am. J. Ophthalmol. 134, 592–596. 10.1016/s0002-9394(02)01642-2 12383817

[B73] Wang Y. Jin Y. Mae M. A. Zhang Y. Ortsater H. Betsholtz C. (2017). Smooth muscle cell recruitment to lymphatic vessels requires PDGFB and impacts vessel size but not identity. Development 144, 3590–3601. 10.1242/dev.147967 28851707 PMC5665477

[B74] Wigle J. T. Oliver G. (1999). Prox1 function is required for the development of the murine lymphatic system. Cell 98, 769–778. 10.1016/s0092-8674(00)81511-1 10499794

[B75] Wong B. W. Wang X. Zecchin A. Thienpont B. Cornelissen I. Kalucka J. (2017). The role of fatty acid beta-oxidation in lymphangiogenesis. Nature 542, 49–54. 10.1038/nature21028 28024299

[B76] Yamazaki T. Yoshimatsu Y. Morishita Y. Miyazono K. Watabe T. (2009). COUP-TFII regulates the functions of Prox1 in lymphatic endothelial cells through direct interaction. Genes 14, 425–434. 10.1111/j.1365-2443.2008.01279.x 19210544

[B77] Yang Y. Garcia-Verdugo J. M. Soriano-Navarro M. Srinivasan R. S. Scallan J. P. Singh M. K. (2012). Lymphatic endothelial progenitors bud from the cardinal vein and intersomitic vessels in mammalian embryos. Blood 120, 2340–2348. 10.1182/blood-2012-05-428607 22859612 PMC3447786

[B78] Yang Y. Oliver G. (2014). Development of the mammalian lymphatic vasculature. J. Clin. Invest. 124, 888–897. 10.1172/JCI71609 24590273 PMC3938267

[B79] Yoo H. Lee Y. J. Park C. Son D. Choi D. Y. Park J. H. (2020). Epigenetic priming by Dot1l in lymphatic endothelial progenitors ensures normal lymphatic development and function. Cell Death Dis. 11, 14. 10.1038/s41419-019-2201-1 31908356 PMC6944698

[B80] Yoshimatsu Y. Yamazaki T. Mihira H. Itoh T. Suehiro J. Yuki K. (2011). Ets family members induce lymphangiogenesis through physical and functional interaction with Prox1. J. Cell Sci. 124, 2753–2762. 10.1242/jcs.083998 21807940

